# Functional Mitral Regurgitation Trajectories in CRT-Treated HFrEF During Medical Therapy Optimization: Association with Reverse Remodeling

**DOI:** 10.3390/jcm15197688

**Published:** 2026-10-04

**Authors:** Oana Pătru, Silvia Luca, Dragoș Cozma, Bogdan Enache, Liviu Cirin, Mirela Virtosu, Maria-Laura Crăciun, Constantin-Tudor Luca, Simona Ruxanda Drăgan

**Affiliations:** 1 Cardiology Department, “Victor Babes” University of Medicine and Pharmacy, 300041 Timisoara, Romania; oana.patru@umft.ro (O.P.); dragos.cozma@umft.ro (D.C.); bogdan.enache@umft.ro (B.E.); laura.craciun@umft.ro (M.-L.C.); constantin.luca@umft.ro (C.-T.L.); simona.dragan@umft.ro (S.R.D.); 2 Research Center of the Institute of Cardiovascular Diseases Timisoara, 13A Gheorghe Adam Street, 300310 Timisoara, Romania; 3 Doctoral School, “Victor Babes” University of Medicine and Pharmacy, 300041 Timisoara, Romania; cirin.liviu@umft.ro (L.C.); daniela.cozma@umft.ro (M.V.); 4 Institute of Cardiovascular Diseases Timisoara, 13A Gheorghe Adam Street, 300310 Timisoara, Romania; 5 Department of Internal Medicine I, “Victor Babes” University of Medicine and Pharmacy, 2 Eftimie Murgu Sq., 300041 Timisoara, Romania

**Keywords:** heart failure with reduced ejection fraction, functional mitral regurgitation, cardiac resynchronization therapy, sacubitril/valsartan, angiotensin receptor–neprilysin inhibitor, reverse remodeling, left ventricular remodeling, echocardiography

## Abstract

**Background:** Functional mitral regurgitation (MR) is common in patients with heart failure with reduced ejection fraction (HFrEF) and may persist despite cardiac resynchronization therapy (CRT). Sacubitril/valsartan is associated with reverse remodeling in HFrEF, but MR dynamics in CRT-treated patients receiving sacubitril/valsartan remain insufficiently characterized. We aimed to characterize longitudinal changes in MR severity and assess their association with ventricular reverse remodeling in CRT-treated patients with HFrEF receiving sacubitril/valsartan. **Methods:** This retrospective cohort study included 188 patients with HFrEF previously treated with CRT who subsequently initiated sacubitril/valsartan. Clinical and echocardiographic assessments were performed at baseline and 10–14 months. The primary endpoint was MR improvement, defined a priori as a reduction of at least one MR grade. Changes in paired MR grade distribution were assessed using Bowker’s test of symmetry. Associations between MR improvement and changes in left ventricular ejection fraction (LVEF), left ventricular end-diastolic volume (LVEDV), and left atrial volume (LAV) were evaluated. Multivariable logistic regression was used to identify factors independently associated with MR improvement. **Results:** MR improvement occurred in 116 patients (62%), while 72 (38%) had unchanged grades; no worsening was documented in the paired assessments. A significant shift toward lower MR grades was observed between baseline and follow-up (χ^2^ = 116.0, df = 5, *p* < 0.001). The proportion meeting the endpoint increased from 0% in baseline grade I to 51.2%, 80.0%, and 100% in grades II, III, and IV, respectively. Patients with MR improvement had greater increases in LVEF (9.0 vs. 7.0 percentage points, *p* = 0.006) and larger reductions in LVEDV (−45.7 vs. −28.6 mL, *p* = 0.004) and LAV (−20.4 vs. −4.2 mL, *p* < 0.001). The correlation between changes in LVEDV and MR grade was statistically significant but modest (*r* = 0.22, *p* = 0.002). Non-ischemic etiology and greater baseline LVEDV were independently associated with MR improvement, whereas baseline LVEF was not. The strong association with baseline MR grade partly reflects the endpoint’s inherent dependence on initial severity. **Conclusions:** In patients with HFrEF treated with CRT who also received sacubitril/valsartan, lower MR grades were observed at follow-up and were associated with greater longitudinal changes in LVEF, LVEDV, and LAV. These findings describe parallel longitudinal changes in MR severity and cardiac structure within a combined device- and pharmacological-treatment setting. The observed changes cannot be disentangled from ongoing CRT-mediated remodeling, concomitant GDMT, changes in loading conditions, or other time-dependent factors and cannot be attributed specifically to sacubitril/valsartan.

## 1. Introduction

Functional mitral regurgitation (MR) is a common finding in patients with heart failure with reduced ejection fraction (HFrEF) and is closely related to left ventricular remodeling and geometric distortion of the mitral apparatus. Its presence is associated with worse symptoms, reduced functional capacity, and adverse prognosis. Beyond its role as a marker of disease severity, functional MR may contribute to volume overload, progressive ventricular dilation, and further deterioration of cardiac function, potentially creating a self-perpetuating cycle of heart failure progression [1,2].

In patients treated with cardiac resynchronization therapy (CRT), improvement in ventricular synchrony may reduce MR severity through favorable changes in ventricular geometry and papillary muscle coordination. By restoring coordinated contraction, CRT can reduce leaflet tethering and improve coaptation. However, despite optimal device implantation and adequate biventricular pacing, persistent or incompletely improved MR remains common [3]. This residual regurgitation remains clinically relevant, as it is associated with ongoing symptoms, reduced response to CRT, and worse long-term outcomes [2].

Previous studies have reported associations between sacubitril/valsartan therapy and reverse remodeling in patients with HFrEF, including improvements in left ventricular systolic function and reductions in ventricular volumes [4,5]. These observations have been linked to favorable neurohormonal and hemodynamic changes [6]. Changes in ventricular structure may also occur in parallel with changes in functional MR [7,8]. However, MR dynamics in CRT-treated patients receiving sacubitril/valsartan remain insufficiently characterized [9,10,11].

Functional MR is primarily considered a consequence of ventricular disease rather than a primary valvular abnormality. Alterations in ventricular size, shape, and function lead to displacement of the papillary muscles and distortion of the mitral valve apparatus, resulting in incomplete leaflet coaptation [1]. In this context, therapies that reverse ventricular remodeling have the potential to improve MR severity even in the absence of direct valve intervention [7]. The interplay between device-based therapies, such as CRT, and pharmacological therapies, such as angiotensin receptor–neprilysin inhibition (ARNI), may therefore be important in determining the overall evolution of MR.

From a clinical standpoint, the management of persistent functional MR in patients with HFrEF remains challenging. While transcatheter and surgical mitral valve interventions have emerged as treatment options in selected patients, current guidelines emphasize the importance of optimizing GDMT before considering invasive approaches [12]. However, longitudinal MR dynamics in CRT-treated patients receiving sacubitril/valsartan and their relationship with ventricular remodeling remain insufficiently characterized.

Patients treated with CRT therefore represent a clinically important group in whom persistent functional MR may continue to evolve during ongoing medical therapy. Characterizing MR trajectories in this setting may provide further insight into the relationship between valvular changes and ventricular remodeling before consideration of subsequent interventions.

The aim of the present study was to characterize longitudinal changes in functional MR severity and assess their association with ventricular reverse remodeling in patients with HFrEF treated with CRT who also received sacubitril/valsartan.

## 2. Materials and Methods

### 2.1. Study Design and Population

The study was conducted as a retrospective cohort analysis at a tertiary cardiovascular center and included patients with HFrEF who had previously undergone CRT and were subsequently initiated on sacubitril/valsartan therapy.

Eligible patients were adults with established HFrEF (left ventricular ejection fraction ≤40%) and persistent systolic dysfunction despite CRT. All patients had stable device function with effective biventricular pacing prior to ARNI initiation. Inclusion required the availability of complete clinical and echocardiographic data at both baseline and follow-up. Patients with significant primary valvular disease requiring intervention, congenital cardiac abnormalities, suboptimal CRT performance, or incomplete imaging data were excluded. A screening log of all potentially eligible patients, including those without follow-up assessments, was not available in the analytical dataset.

Baseline (T0) was defined as the time of sacubitril/valsartan initiation, while follow-up (T1) corresponded to 10–14 months thereafter. The interval between CRT implantation and ARNI initiation varied among patients, reflecting differences in clinical course; however, all analyses were anchored to ARNI initiation as a uniform reference point.

Clinical and demographic data, including age, sex, heart failure etiology, NYHA functional class, and pharmacological therapy, were obtained from electronic medical records. The study protocol was approved by the institutional ethics committee and conducted in accordance with the principles of the Declaration of Helsinki.

The primary outcome was improvement in MR severity, defined as a reduction of at least one MR grade between baseline and follow-up.

### 2.2. Medical Therapy and Device Treatment

All patients received guideline-directed medical therapy (GDMT) according to contemporary heart failure recommendations. Beta-blockers were prescribed and titrated to the maximally tolerated dose whenever clinically feasible, while treatment was adjusted as needed to maintain a high proportion of effective biventricular pacing [13]. Mineralocorticoid receptor antagonists were prescribed whenever tolerated. Sodium–glucose cotransporter-2 inhibitors were used in the majority of patients and were generally stable prior to ARNI initiation.

Sacubitril/valsartan was initiated during hospitalization, either in clinically stable patients undergoing routine treatment optimization or after stabilization following an episode of acute heart failure decompensation. In patients admitted with acute congestion, treatment initiation occurred after effective decongestion had been achieved. The initial sacubitril/valsartan dose (24/26 mg or 49/51 mg twice daily) was selected according to blood pressure, renal function, serum potassium levels, and prior treatment with angiotensin-converting enzyme inhibitors or angiotensin receptor blockers. The dose was titrated toward the maximally tolerated dose during outpatient follow-up visits at approximately 4–8-week intervals. The tolerated maintenance dose was recorded at follow-up, and follow-up echocardiography was performed at 10–14 months.

Patients were treated with CRT systems including CRT-P, CRT-D, or left ventricular pacing configurations with right atrial and left ventricular leads, all providing effective resynchronization therapy. Device function and biventricular pacing were assessed during routine follow-up. For patients treated with left ventricular fusion pacing configurations, device optimization and assessment of effective resynchronization followed established principles of LV-only fusion CRT pacing [14].

Loop diuretic therapy was recorded at baseline and follow-up, and changes in dose were evaluated as part of the clinical assessment.

Management and follow-up reflected routine clinical practice, including outpatient visits and device evaluations.

### 2.3. Echocardiographic Assessment

Transthoracic echocardiographic examinations were performed at both study time points in the same echocardiography laboratory, following standardized acquisition protocols and current ASE/EACVI recommendations. Imaging was performed using VIVID echocardiographic systems (GE Healthcare, Milwaukee, WI, USA) equipped with a 2.5-MHz transducer.

The baseline echocardiographic assessment was performed at the time of sacubitril/valsartan initiation, whereas the follow-up examination was scheduled at 10–14 months after treatment initiation. In patients hospitalized with acute congestion, baseline echocardiography was performed after clinical stabilization and effective decongestion, rather than during the acute congestive phase. Left ventricular ejection fraction (LVEF) was determined using the biplane Simpson method. Left ventricular end-diastolic volume (LVEDV) was obtained from standard apical views, while left atrial volume (LAV) was measured at end-systole in accordance with established echocardiographic recommendations.

MR severity was extracted from routine clinical echocardiography reports available in the patients’ medical records and classified on a four-grade scale (I–IV), corresponding to mild, moderate, moderate-to-severe, and severe regurgitation. No retrospective reassessment of the original images was performed, and the original clinical assessments were not blinded to clinical information or examination time point. The individual echocardiographic parameters underlying the reported grades were not systematically available in the extracted dataset, and quantitative measures, including effective regurgitant orifice area (EROA) and regurgitant volume, were unavailable for analysis. Accordingly, longitudinal MR changes were assessed using clinically reported ordinal grades without quantitative confirmation. MR improvement was defined a priori as a reduction of at least one reported MR grade between baseline and follow-up.

The extent of reverse remodeling was assessed by comparing LVEF, LVEDV, and LAV values documented in the clinical echocardiography reports at baseline and follow-up.

### 2.4. Study Endpoints

The primary endpoint was improvement in clinically reported MR severity, defined a priori as a reduction of at least one ordinal MR grade between baseline and follow-up.

Secondary endpoints included: (1) changes in the distribution of MR grades between baseline and follow-up; (2) the association between MR improvement and echocardiographic reverse remodeling, assessed by changes in LVEF, LVEDV, and LAV; (3) the relationship between MR improvement and clinical status, evaluated using changes in NYHA functional class.

Additional exploratory analyses included changes in loop diuretic therapy as an indirect marker of decongestion and clinical improvement.

### 2.5. Statistical Analysis

All statistical analyses were performed using MedCalc Statistical Software version 12.5 (MedCalc Software Ltd., Ostend, Belgium).

Continuous variables are presented as mean ± standard deviation, and categorical variables as counts and percentages. Patients were categorized into two groups according to the presence or absence of MR improvement, defined as a ≥1-grade reduction in MR severity. Between-group comparisons were performed using independent-samples *t*-tests for continuous variables and chi-square or Fisher’s exact tests for categorical variables, as appropriate. For ordinal variables, non-parametric comparisons were used when appropriate.

Changes in MR severity between baseline and follow-up were assessed using paired categorical analysis. Bowker’s test of symmetry was used to assess asymmetry in paired transitions between baseline and follow-up MR grades. This test does not incorporate the ordering of MR categories or quantify the magnitude of improvement. The direction of change was described separately using the observed grade distributions and the proportions of patients with improved, unchanged, or worsened MR.

Associations between reverse remodeling parameters and changes in MR severity were explored using correlation analysis. Variables potentially associated with MR improvement were further evaluated using multivariable logistic regression analysis. The adjusted model included age, sex, cardiomyopathy etiology, baseline LVEF, baseline LVEDV, and baseline MR grade. Baseline MR grade was included as a covariate; however, this adjustment does not eliminate the endpoint’s inherent dependence on baseline severity. Changes in NYHA functional class and loop diuretic therapy were assessed as secondary clinical measures. A two-sided *p*-value < 0.05 was considered statistically significant.

## 3. Results

### 3.1. Study Population and Baseline Characteristics

The analytical cohort comprised 188 patients with HFrEF treated with CRT who subsequently received sacubitril/valsartan and had paired clinical and echocardiographic assessments available at baseline and follow-up (10–14 months).

At follow-up, the tolerated maintenance dose of sacubitril/valsartan, expressed as the total daily dose, was 100 mg/day in 62 patients (33.0%), 200 mg/day in 98 patients (52.1%), and 400 mg/day in 28 patients (14.9%).

Improvement in MR severity by at least one grade was observed in 116 patients (62%), while 72 patients (38%) showed no improvement. No patients experienced worsening of MR during follow-up.

Baseline characteristics differed between patients with and without MR improvement (Table 1). Demographic variables, including age and sex distribution, were comparable between groups. However, significant differences were observed in underlying disease characteristics and cardiac structure.

Patients with MR improvement had a lower baseline LVEF and larger LVEDV than those without MR improvement. In addition, ischemic etiology was more frequent among patients without MR improvement. Baseline NYHA functional class and MR severity also differed between groups, with a higher proportion of moderate-to-severe and severe MR in the improvement group.

These findings show that patients with more severe baseline MR were more likely to meet the predefined criterion for MR improvement, while baseline ventricular structure and function also differed between groups.

### 3.2. Changes in MR Severity

At baseline, MR was grade I in 16 patients (8.5%), grade II in 82 patients (43.6%), grade III in 80 patients (42.6%), and grade IV in 10 patients (5.3%). At follow-up, MR was grade I in 64 patients (34.0%), grade II in 100 patients (53.2%), and grade III in 24 patients (12.8%), while no patients exhibited grade IV MR.

MR severity changed significantly between baseline and follow-up. In the paired analysis, 116 of 188 patients experienced an improvement of at least one MR grade, 72 patients had unchanged MR severity, and no patient demonstrated MR worsening. Bowker’s test indicated significant asymmetry in paired MR grade transitions (χ^2^ = 116.0, df = 5, *p* < 0.001). The observed transitions were toward lower MR grades, with 116 patients improving, 72 remaining unchanged, and none showing worsening.

A marked shift toward lower MR grades was observed at follow-up compared with baseline (Table 2, Figure 1). In particular, the proportion of patients with grade III–IV MR decreased substantially, while the proportion of patients with mild regurgitation increased.

The likelihood of MR improvement varied according to baseline severity. Improvement was observed in 51.2% of patients with grade II MR, 80.0% of patients with grade III MR, and 100.0% of patients with grade IV MR, while no improvement was observed in patients with grade I MR.

These findings show that the likelihood of achieving the predefined ≥1-grade reduction in MR increased with greater baseline MR severity, although this pattern is partly expected from the ordinal definition of the endpoint and the greater room for improvement among patients with higher baseline MR grades.

A patient-level representation of MR dynamics is shown in Figure 2. Most patients were located below the line of identity, indicating a reduction in MR severity between baseline and follow-up. Improvement of at least one grade was observed in 62% of patients, while no cases of MR worsening were identified. These findings illustrate an overall shift toward lower MR grades between baseline and follow-up.

### 3.3. Reverse Remodeling and Clinical Changes According to MR Improvement

Patients meeting the MR-improvement endpoint showed greater longitudinal changes in remodeling parameters than those without MR improvement (Table 3). MR improvement was associated with a greater increase in LVEF and larger reductions in LVEDV and LAV.

Functional status improved in both groups during follow-up; however, the magnitude of improvement was greater among patients with MR reduction. A similar trend was observed for loop diuretic therapy, with dose reduction occurring more frequently in the improvement group, although this difference did not reach statistical significance.

Reduction in LVEDV was modestly correlated with the degree of MR improvement (*r* = 0.22, *p* = 0.002), indicating that greater ventricular volume reduction was associated with greater improvement in MR severity.

Greater reductions in LVEDV were consistently observed in patients with MR improvement (Figure 3), further illustrating the association between ventricular remodeling and changes in MR severity.

### 3.4. Factors Associated with MR Improvement

Factors associated with MR improvement were evaluated using multivariable logistic regression analysis (Table 4). Given that the outcome was defined as a reduction of at least one MR grade, baseline MR severity was included in the adjusted model to account for the differential opportunity to achieve the endpoint across baseline MR grades.

The model included age, sex, cardiomyopathy etiology, baseline LVEF, baseline LVEDV, and baseline MR grade. Baseline MR grade was strongly associated with subsequent MR improvement, with each one-grade increase in baseline MR severity associated with higher odds of meeting the predefined endpoint (OR 4.90, 95% CI 3.13–9.42, *p* < 0.001). However, this association should be interpreted in the context of the endpoint definition, as patients with higher baseline MR grades had greater opportunity to achieve a ≥1-grade reduction. Non-ischemic cardiomyopathy was independently associated with MR improvement (OR 3.53, 95% CI 1.75–7.35, *p* = 0.001), as was baseline LVEDV (OR 1.009 per mL, 95% CI 1.002–1.016, *p* = 0.013). Age, male sex, and baseline LVEF were not independently associated with MR improvement after adjustment.

## 4. Discussion

The present study characterized longitudinal changes in functional MR severity and their association with ventricular remodeling in patients with HFrEF treated with CRT who also received sacubitril/valsartan. The main findings can be summarized as follows: (1) MR severity decreased substantially during follow-up, with an improvement of at least one grade observed in the majority of patients; (2) patients meeting the predefined MR-improvement endpoint also showed greater longitudinal changes in ventricular structure and function; and (3) patients with more advanced ventricular dysfunction and more severe baseline regurgitation were more likely to meet the predefined criterion for MR improvement. To our knowledge, this is one of the first studies specifically characterizing MR dynamics in CRT-treated patients receiving ARNI therapy.

These findings are consistent with the established relationship between functional MR and ventricular remodeling in this population [1]. The observed reduction in MR severity occurred in parallel with improvements in left ventricular function and reductions in ventricular and atrial volumes, consistent with prior observations [4,5]. Importantly, patients who experienced MR improvement showed greater longitudinal changes in remodeling parameters, supporting an association between ventricular and valvular changes.

### 4.1. Association Between MR Evolution and Ventricular Remodeling

Functional MR in HF is primarily driven by geometric alterations of the left ventricle, leading to displacement of papillary muscles, increased leaflet tethering, and impaired coaptation. In this context, the mitral valve itself is structurally normal, and regurgitation reflects an imbalance between closing and tethering forces. Changes in ventricular geometry may therefore influence mitral valve competence [1].

CRT addresses mechanical dyssynchrony and has been shown to reduce MR through improved coordination of papillary muscle contraction and more efficient ventricular systole. However, CRT alone does not fully reverse adverse remodeling in all patients, and residual MR remains common [3].

Ongoing device-mediated and pharmacological remodeling may both contribute to MR evolution in CRT-treated patients, but their individual contributions cannot be separated in the present study. Previous studies have reported associations between ARNI therapy, reductions in ventricular volumes, improvements in systolic function, and changes in functional MR [7,8]. In our cohort, however, the correlation between changes in LVEDV and MR grade was statistically significant but modest (*r* = 0.22). This finding is compatible with a potential contribution of reverse remodeling but does not establish a remodeling-mediated mechanism or demonstrate causality. Changes in loading conditions and variability in clinical MR grading may also contribute to the observed association. Previous observations of remodeling following sacubitril/valsartan initiation in CRT-treated patients provide context for this interpretation but do not establish the mechanism underlying the changes observed in our cohort [9].

### 4.2. Factors Associated with MR Improvement

Higher baseline MR grade was associated with a greater likelihood of meeting the predefined ≥1-grade improvement endpoint. In the multivariable model, each one-grade increase in baseline MR severity was associated with approximately five-fold higher odds of achieving this endpoint. However, this association is partly inherent to the endpoint definition: patients with grade I MR cannot improve by this criterion, whereas those with grades III–IV have greater opportunity to achieve a reduction of at least one grade. Adjustment for other baseline covariates does not remove this structural dependence. Baseline MR grade should therefore be interpreted as a factor associated with meeting the defined ordinal endpoint, rather than as evidence of greater biological responsiveness or treatment benefit. Regression to the mean and variability in clinical MR grading may further contribute to this association.

Baseline LVEDV was also independently associated with MR improvement, although the magnitude of the association was modest. This finding is consistent with the established relationship between ventricular remodeling and functional MR, whereby changes in ventricular size and geometry may influence mitral valve competence [1,4,7,8]. In contrast, baseline LVEF was not independently associated with MR improvement after adjustment for baseline MR grade, LVEDV, age, sex, and cardiomyopathy etiology. Non-ischemic etiology was also independently associated with a higher likelihood of MR improvement. The association between non-ischemic etiology and MR improvement may also reflect differences in responsiveness to CRT and underlying myocardial substrate, rather than a differential response to sacubitril/valsartan. The present design cannot distinguish between these potential explanations.

The progressive increase in the proportion of patients achieving the predefined endpoint across baseline MR grades should therefore not be interpreted as evidence that patients with more severe MR necessarily derive greater therapeutic benefit. Rather, this pattern likely reflects both the greater potential for measurable improvement at higher baseline grades and the association between MR severity and the underlying ventricular disease [1,2]. These observations characterize factors associated with the predefined MR-change endpoint, although prospective studies with standardized MR assessment are required before they can be used to inform therapeutic expectations.

### 4.3. Clinical Implications

From a clinical perspective, persistent functional MR is frequently encountered in patients treated with CRT and remains associated with ongoing symptoms and adverse outcomes [2,3]. In the present cohort, lower MR grades were frequently observed during follow-up in patients receiving both CRT and contemporary GDMT, including sacubitril/valsartan.

The observed association between MR changes and ventricular remodeling should be interpreted within the current clinical framework, in which GDMT is optimized before consideration of mitral valve intervention [1,2,12]. Assessment of ventricular remodeling and MR evolution may therefore provide useful additional information when evaluating longitudinal clinical and imaging changes.

Improvements in NYHA functional class and the non-significant trend toward reduced loop diuretic requirements were directionally consistent with the echocardiographic findings. Although these clinical changes were exploratory, they illustrate the potential value of integrating imaging and clinical parameters when characterizing longitudinal changes in patient status [15,16,17].

In clinical practice, CRT and GDMT are used concurrently in patients with HFrEF. The present findings therefore reflect a combined device- and pharmacological-treatment setting rather than the isolated effect of either therapeutic component [4,9,10,11].

### 4.4. Comparison with Previous Studies

Previous studies have consistently reported associations between sacubitril/valsartan therapy, reverse remodeling, and improvements in left ventricular function in patients with HFrEF [4,5,6,17,18,19,20]. Similar findings have also been reported in CRT-treated populations, including patients with suboptimal response to resynchronization therapy [9,10]. Sardu et al. reported that sacubitril/valsartan was associated with favorable remodeling and clinical improvement in CRT-D non-responders, providing relevant context for remodeling observed in patients receiving both CRT and pharmacological therapy [21]. Several investigations have suggested an association between ARNI therapy and improvement in functional MR, although these studies have generally included heterogeneous populations and have not specifically focused on CRT-treated patients [2,7,8]. CRT itself is also known to influence functional MR through improved ventricular synchrony, papillary muscle coordination, and favorable changes in ventricular geometry [3]. Nevertheless, residual MR may persist despite CRT, particularly in patients with incomplete reverse remodeling or limited response to resynchronization therapy [3,9,10].

Additional imaging approaches may help characterize the mechanical substrate underlying CRT response. One study reported that gated single-photon emission computed tomography (G-SPECT) can quantify left ventricular mechanical dyssynchrony and that selected phase-analysis parameters predicted LVEF normalization following CRT [22]. These findings suggest a potential complementary role for G-SPECT in the assessment of CRT candidates and highlight the relevance of mechanical dyssynchrony to subsequent ventricular recovery. However, they do not establish a relationship with MR improvement or distinguish device-related from pharmacological remodeling in the present cohort.

The present study adds to the existing literature by characterizing longitudinal MR dynamics in patients with HFrEF treated with CRT who also received sacubitril/valsartan. Whereas previous work has primarily focused on reverse remodeling, clinical response, or CRT non-response, our analysis specifically evaluated changes in MR severity and their association with ventricular remodeling. These observations extend the available evidence by describing how MR evolution relates to changes in ventricular structure and function in this combined treatment setting.

Because all patients in the present cohort received both CRT and sacubitril/valsartan, the observed changes occurred within a combined device- and pharmacological-treatment setting. The variable interval between CRT implantation and ARNI initiation also means that patients may have been at different stages of CRT-related remodeling at baseline. The present findings should therefore be interpreted as describing longitudinal MR evolution and its association with ventricular remodeling in a CRT-treated population receiving sacubitril/valsartan, rather than as an estimate of the independent contribution of either treatment modality. Accordingly, the observed longitudinal changes should also be interpreted in the context of concomitant GDMT, potential changes in loading conditions, and the selection and survivorship bias inherent to the paired-analysis cohort.

### 4.5. Limitations

The retrospective single-center design limits generalizability and introduces potential selection bias. The analytical cohort was assembled directly from patients with available paired baseline and follow-up echocardiographic assessments. Although echocardiography was routinely performed and documented for patients returning for hospitalization, patients who died before reassessment or did not return to the center would not be represented in the paired analysis. The number and characteristics of potentially eligible patients without follow-up imaging could not be determined from the available analytical dataset, limiting assessment of the magnitude and direction of selection and survivorship bias. Consequently, the absence of observed MR worsening applies only to the selected cohort with paired assessments and should not be generalized to all patients initiating sacubitril/valsartan.

All patients received sacubitril/valsartan, and no untreated comparator group was available; therefore, the study cannot establish a causal effect of ARNI therapy on MR improvement. Treatment strategies and follow-up assessments were not standardized by a prospective protocol, and residual confounding cannot be excluded. Changes in MR severity may have been influenced by the natural course of HF, potential adjustments to other components of GDMT during follow-up, changes in loading conditions, and ongoing CRT-mediated remodeling. In particular, the variable interval between CRT implantation and ARNI initiation means that patients may have been at different stages of their remodeling trajectory at baseline. In addition, the degree of clinical and echocardiographic response achieved during the period between CRT implantation and ARNI initiation was not systematically characterized in the analytical dataset. These factors limit the ability to separate the contributions of device-based and pharmacological therapy.

MR severity was assessed using a semi-quantitative ordinal grading system, with grades extracted from routine clinical echocardiography reports. Although examinations were performed within the same laboratory using consistent clinical protocols, no retrospective image reassessment or independent core-laboratory adjudication was performed, and the original assessments were not blinded to clinical information or examination time point. Interobserver variability, assessment bias, and differences in loading conditions may therefore have influenced the reported grades. Quantitative measures, including effective regurgitant orifice area (EROA) and regurgitant volume, were unavailable, precluding quantitative confirmation of longitudinal changes in MR severity. LVESVi, an established parameter of ventricular reverse remodeling and CRT response, was not available in the analytical dataset and could therefore not be included in the analysis.

The ≥1-grade improvement endpoint is inherently dependent on baseline MR severity, creating a floor effect for patients with grade I MR and greater opportunity to meet the endpoint at higher grades. This limits the interpretation of baseline MR grade as a biologically meaningful factor associated with improvement, even after multivariable adjustment. Furthermore, although statistically significant, the association between changes in LVEDV and MR grade was modest (*r* = 0.22) and should not be interpreted as evidence that reverse remodeling mediated the observed MR improvement.

Natriuretic peptide levels were not systematically available, limiting the ability to relate imaging findings to biochemical markers of heart failure severity. Changes in loop diuretic therapy were evaluated as an exploratory clinical marker but may have been influenced by factors other than MR improvement.

Finally, follow-up was limited to approximately one year, and the long-term durability of MR improvement was not assessed. Subsequent mitral valve interventions, hospitalization, and survival were not evaluated as study outcomes; therefore, the clinical implications of the observed echocardiographic and functional changes remain uncertain.

### 4.6. Future Perspectives

Further studies are warranted to better characterize the factors associated with longitudinal changes in functional MR in patients receiving combined device-based and pharmacological therapy. Prospective studies incorporating quantitative echocardiographic assessment of MR, standardized assessment of loading conditions, and more detailed evaluation of ventricular geometry may provide additional insight into the factors associated with MR evolution and valve competence.

Longer-term follow-up studies are needed to assess the persistence of longitudinal changes in MR severity and determine whether these changes are associated with clinically relevant outcomes, including heart failure hospitalization, functional status, and survival.

Future research should also evaluate whether changes in MR severity observed during GDMT optimization are associated with the timing or need for subsequent mitral valve intervention, particularly among patients initially considered for transcatheter or surgical treatment. Prospective comparative studies with appropriate control groups will be important to distinguish treatment-related effects from the natural evolution of heart failure and the effects of CRT and concomitant medical therapy.

## 5. Conclusions

In patients with HFrEF treated with CRT who also received sacubitril/valsartan, MR severity shifted toward lower grades between baseline and follow-up, with improvement of at least one grade observed in 62% of patients. MR improvement was associated with greater changes in ventricular structure and function, including larger increases in LVEF and greater reductions in LVEDV and LAV. In multivariable analysis, non-ischemic etiology and greater baseline LVEDV were independently associated with MR improvement, whereas baseline LVEF was not. More severe baseline MR was strongly associated with meeting the predefined one-grade improvement endpoint; however, this finding should be interpreted in the context of the greater opportunity for categorical improvement among patients with higher baseline MR grades.

These findings describe a longitudinal association between MR evolution and changes in ventricular structure and function in a CRT-treated population receiving contemporary GDMT, including sacubitril/valsartan. The observed changes occurred within a combined device- and pharmacological-treatment setting and cannot be disentangled from ongoing CRT-related remodeling, concomitant therapies, changes in loading conditions, selection and survivorship bias, and other time-dependent factors. Accordingly, the present observational design does not permit estimation of an independent or incremental effect of sacubitril/valsartan beyond CRT. From a clinical perspective, these findings should be interpreted within the current management framework in which both device therapy and guideline-directed medical therapy are optimized in patients with HFrEF and persistent functional MR.

## Figures and Tables

**Figure 1:**
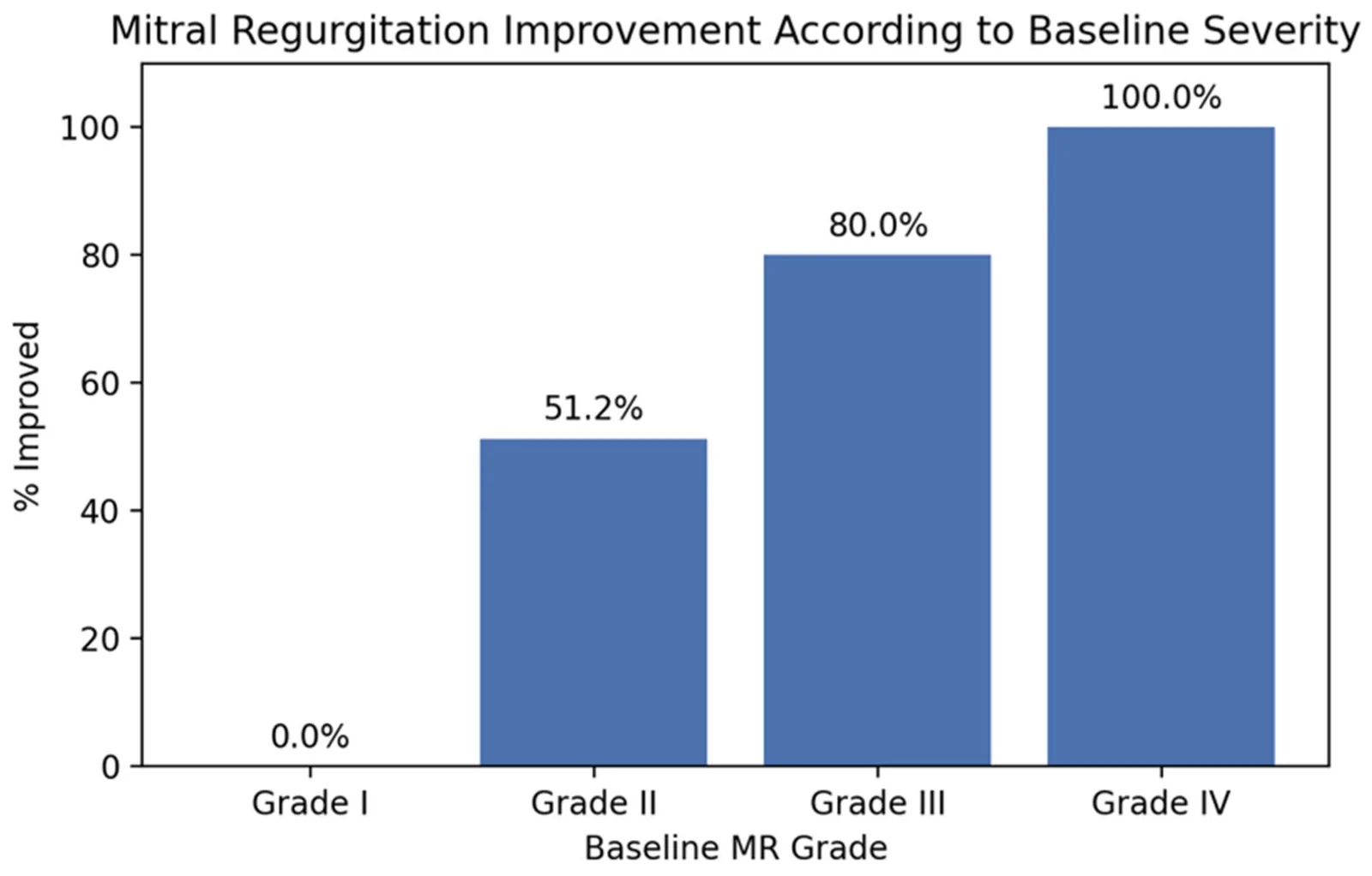
MR improvement according to baseline severity. Bar chart showing the proportion of patients achieving ≥1-grade reduction in MR severity according to baseline MR grade. The proportion of patients meeting the predefined criterion for improvement increased progressively with baseline MR severity, with no improvement observed among patients with grade I MR and the highest proportions observed among patients with grade III and IV MR.

**Figure 2:**
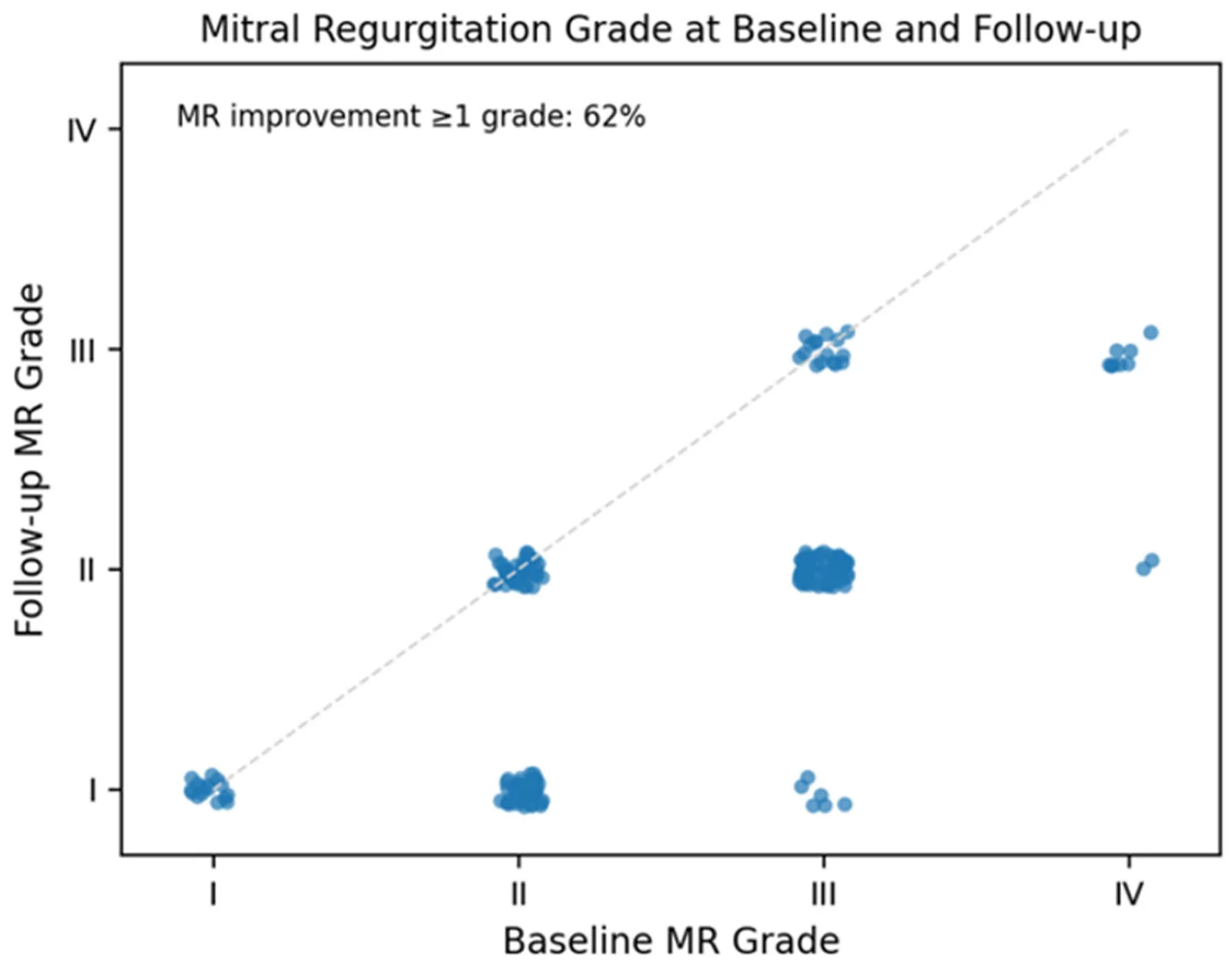
Individual changes in MR severity from baseline to follow-up. Scatter plot showing MR grade at baseline (*x*-axis) and follow-up (*y*-axis) for individual patients. The dashed diagonal line represents no change in MR severity. Points below the line indicate improvement, while points above indicate worsening. The majority of patients demonstrated a reduction in MR grade, with overall improvement of ≥1 grade observed in 62% of the cohort and no cases of worsening.

**Figure 3:**
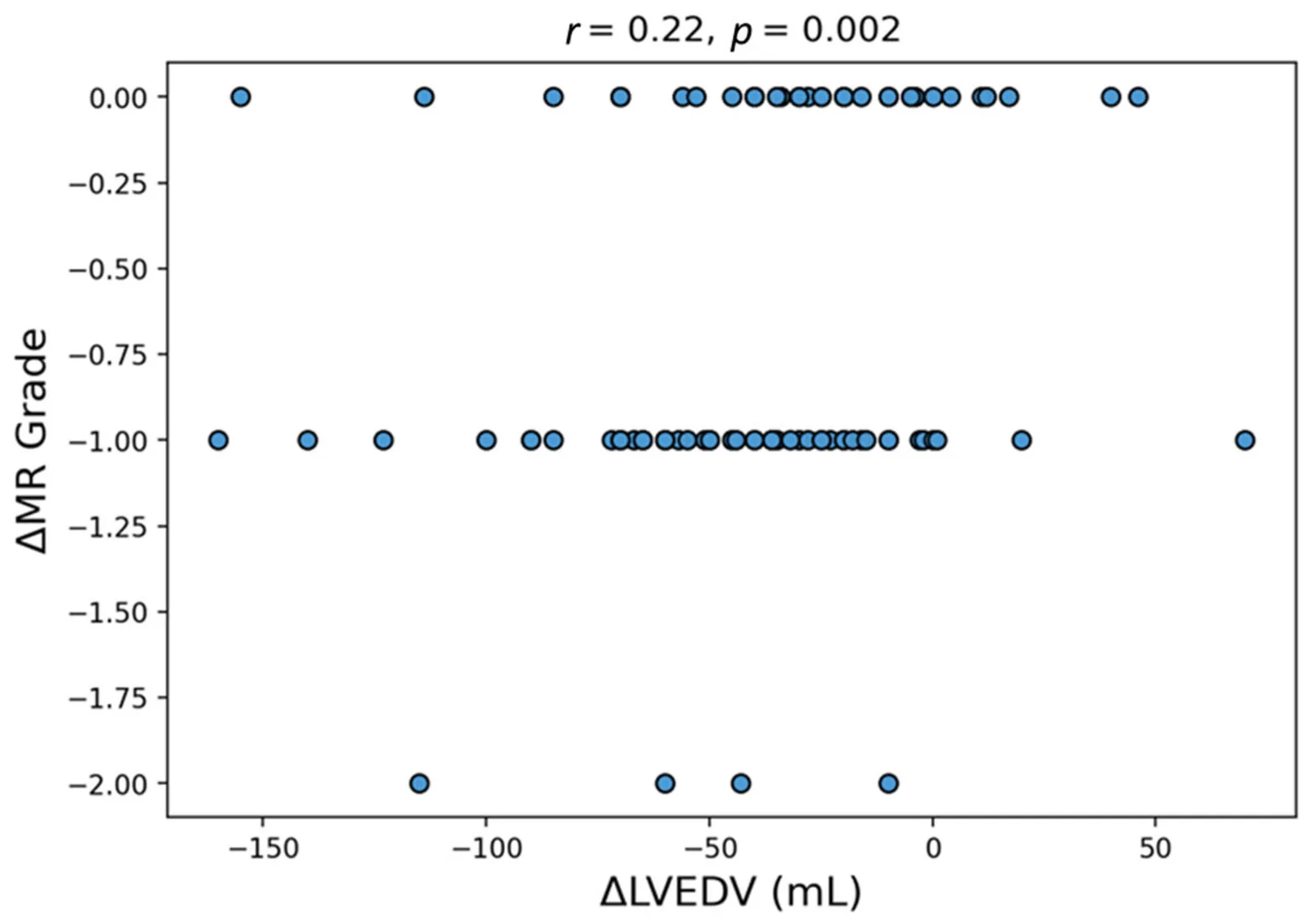
Relationship between left ventricular reverse remodeling and MR improvement. Scatter plot illustrating the association between change in LVEDV (ΔLVEDV) and change in MR grade between baseline and follow-up. Each blue circle represents an individual patient. Negative values of ΔLVEDV indicate reduction in ventricular volume (reverse remodeling), while negative values of ΔMR grade indicate improvement in MR severity. A significant positive correlation was observed (*r* = 0.22, *p* = 0.002), indicating that greater reductions in LVEDV were associated with greater improvement in MR severity.

**Table 1:** Baseline characteristics according to MR improvement.

Variable	MR Improved (*n* = 116)	No MR Improvement (*n* = 72)	*p*-Value
Age (years)	68.6 ± 9.7	66.9 ± 9.2	0.228
Sex	Male: 72 (62%) Female: 44 (38%)	Male: 48 (67%) Female: 24 (33%)	0.537
Cardiomyopathy etiology	ischemic: 28 (24%) nonischemic: 88 (76%)	ischemic: 32 (44%) Nonischemic: 40 (56%)	0.006
LVEF (%)	33.2 ± 6.4	35.3 ± 5.9	<0.001
LVEDV (mL)	225.5 ± 79.6	191.2 ± 47.7	<0.001
LAV (mL)	92.9 ± 31.3	90.9 ± 29.5	0.975
NYHA class	II: 40 (34.5%) III: 75 (64.7%) IV:1 (0.8%)	II: 40 (55.6%) III: 32 (44.4%) IV: 0 (0.0%)	0.012
MR grade	I: 0 (0.0%) II: 42 (36%) III: 64 (55%) IV: 10 (9%)	I: 16(22%) II: 40 (56%) III: 16 (22%) IV: 0 (0.0%)	<0.001

**Table 2:** MR distribution at baseline and follow-up.

MR Grade	Baseline (T0)	Follow-Up (T1)
Grade I	16 (8.5%)	64 (34.0%)
Grade II	82 (43.6%)	100 (53.2%)
Grade III	80 (42.6%)	24 (12.8%)
Grade IV	10 (5.3%)	0 (0.0%)

**Table 3:** Echocardiographic and clinical changes according to MR improvement.

Parameter	MR Improved (*n* = 116)	No MR Improvement (*n* = 72)	*p*-Value
ΔLVEF (%)	9.0 ± 3.9	7.0 ± 5.2	0.006
ΔLVEDV (mL)	−45.7 ± 38.2	−28.6 ± 39.2	0.004
ΔLAV (mL)	−20.4 ± 25.0	−4.2 ± 23.7	<0.001
ΔNYHA class	−1.11 ± 0.45	−0.94 ± 0.33	0.004
Loop diuretic reduction (patient number)	76 (65.5%)	42 (58.3%)	0.322

**Table 4:** Factors associated with MR improvement.

Variable	OR	95% CI	*p*-Value
Age (per year)	1.03	0.99–1.08	0.171
Male sex	0.67	0.35–1.30	0.239
Non-ischemic etiology	3.53	1.75–7.35	0.001
Baseline LVEF (per 1%)	1.02	0.94–1.10	0.689
Baseline LVEDV (per 1 mL)	1.009	1.002–1.016	0.013
Baseline MR grade (per one-grade increase)	4.90	3.13–9.42	<0.001

## Data Availability

The original contributions presented in this study are included in the article. Further inquiries can be directed to the corresponding author.

## Abbreviations

The following abbreviations are used in this manuscript:

ACE	Angiotensin-converting enzyme
ARB	Angiotensin receptor blocker
ARNI	Angiotensin receptor–neprilysin inhibitor
ASE	American Society of Echocardiography
CI	Confidence interval
CRT	Cardiac resynchronization therapy
CRT-D	Cardiac resynchronization therapy with defibrillator
CRT-P	Cardiac resynchronization therapy with pacemaker
DCM	Dilated cardiomyopathy
E/A	Ratio of early to late diastolic transmitral flow velocities
EACVI	European Association of Cardiovascular Imaging
EROA	Effective regurgitant orifice area
GDMT	Guideline-directed medical therapy
G-SPECT	Gated single-photon emission computed tomography
HF	Heart failure
HFrEF	Heart failure with reduced ejection fraction
IQR	Interquartile range
LAV	Left atrial volume
LV	Left ventricular
LVEDV	Left ventricular end-diastolic volume
LVEF	Left ventricular ejection fraction
MR	Mitral regurgitation
NYHA	New York Heart Association
OR	Odds ratio
SD	Standard deviation
SGLT2 inhibitor	Sodium–glucose cotransporter-2 inhibitor
SPECT	Single-photon emission computed tomography
